# Drug-Induced Complete Atrioventricular Block in an Elderly Patient: A Case Report Highlighting Digoxin-Beta Blocker Interactions and a Paradoxical State

**DOI:** 10.3390/life15020215

**Published:** 2025-01-31

**Authors:** Cristiana Bustea, Andrei-Flavius Radu, Cosmin Mihai Vesa, Ada Radu, Teodora Maria Bodog, Ruxandra Florina Bodog, Paula Bianca Maghiar, Adrian Marius Maghiar

**Affiliations:** 1 Department of Preclinical Disciplines, Faculty of Medicine and Pharmacy, University of Oradea, 410073 Oradea, Romania; bustea@uoradea.ro; 2 Doctoral School of Biological and Biomedical Sciences, University of Oradea, 410087 Oradea, Romania; adaradu@uoradea.ro (A.R.); bodog.teodoramaria@student.uoradea.ro (T.M.B.); bodog.ruxandraflorina@student.uoradea.ro (R.F.B.); marius.maghiar@didactic.uoradea.ro (A.M.M.); 3 Department of Pharmacy, Faculty of Medicine and Pharmacy, University of Oradea, 410028 Oradea, Romania; 4 Department of Surgical Disciplines, Faculty of Medicine and Pharmacy, University of Oradea, 410073 Oradea, Romania; badea.paula.bianca@didactic.uoradea.ro

**Keywords:** atrioventricular block, drug–drug interaction, digoxin toxicity, beta-blocker, hyperkalemia, online drug interaction checker

## Abstract

Complete atrioventricular (AV) block is a severe conduction abnormality caused by intrinsic cardiac disease, ischemia, electrolyte imbalances, or drug interactions. Elderly patients on multiple medications are particularly vulnerable to polypharmacy-related interactions. This case report describes an 82-year-old female presenting to the emergency department with fatigue, syncope, and disorientation. Her medical history included atrial fibrillation, hypertension, and heart failure, with a medication regimen of digoxin 0.25 mg given daily 5 days out of 7, metoprolol 50 mg twice daily, lisinopril 10 mg daily, furosemide 40 mg daily, and spironolactone 50 mg daily. Clinical examination revealed bradycardia and a holosystolic murmur in the mitral valve area, while the electrocardiogram showed complete AV block at a ventricular rate of 35 bpm. Laboratory results indicated mild hyperkalemia (4.9 mmol/L). Suspecting a digoxin–beta-blocker interaction, antiarrhythmic therapy was discontinued. Within three days, the AV block resolved, transitioning to atrial fibrillation with a high ventricular rate. Bisoprolol was introduced for rate control, and hemodynamic stability was achieved. The patient was discharged with a revised medication regimen and showed no recurrence of AV block. This case emphasizes the importance of recognizing drug interactions as a reversible cause of AV block and using drug interaction checkers to manage polypharmacy, especially in elderly patients with multiple comorbidities. It also highlights the rare and paradoxical combination of atrial flutter and complete AV block.

## 1. Introduction

Atrioventricular (AV) block, commonly referred to as heart block, emerges when the partial or complete interruption of the electrical signals originating in the sinoatrial (SA) node prevents them from properly reaching the AV node [1,2]. This blockage may result from either anatomical or functional abnormalities within the heart’s conduction pathways [3,4].

Complete AV or heart block (CHB) or third-degree AV block represents the most severe and pathological form of conduction disturbance, where the electrical signals from the SA node are entirely obstructed from reaching the ventricles. This interruption causes the atria and ventricles to operate independently, with the ventricles relying on an alternative rhythm, either from the His bundle or the ventricular muscle itself, depending on the level of the block. This form of block is generally the end result of either Mobitz type I or type II AV block [2,5]. While first-degree AV block is relatively common, third-degree AV block is rare in healthy adults. However, its prevalence increases significantly with age [6].

The progression to CHB can be triggered by various underlying conditions, with coronary ischemia being the most prevalent [7]. Additionally, acute myocardial infarction, especially in the inferior wall, may result in damage to the AV node, causing a CHB [8]. Moreover, a third-degree AV block can also have congenital origins, with systemic lupus erythematosus being a significant risk factor [9]. Other contributing factors include hyperkalemia, which can impair conduction through the His-Purkinje system, as well as *Borrelia burgdorferi* infection, which may damage the heart via Lyme disease [10,11]. Surgical procedures such as open-heart surgery, percutaneous coronary interventions, or septal alcohol infusion may also lead to AV block [12]. The next few decades are expected to see a significant rise in the occurrence of this condition, making it an important area of focus for future cardiovascular health research and management [13].

In order to enhance the management of complex pathologies, numerous researchers worldwide are interested in comprehending the toxicities caused by drugs and chemicals [14]. Furthermore, in the management of cardiovascular patients, a crucial aspect is the evaluation of medications, especially in cases involving polypharmacy. Many commonly prescribed drugs have the potential to either initiate or worsen arrhythmias. These include antiarrhythmic drugs, antibiotics, psychotropic agents, methadone, and increasingly, medications from various other therapeutic classes, such as neurological drugs, cancer treatments, and more. These drugs can contribute to prolonged QT intervals, increasing the risk of torsades de pointes, a potentially fatal arrhythmia [15,16].

In addition to these risks, drug-induced arrhythmias, particularly bradyarrhythmias, can pose significant dangers, including sudden cardiac death. Some medications may suppress the sinus node, leading to issues such as sinus bradycardia (i.e., heart rate under 60 bpm), sinus pauses, or even sinus arrest. This group of drugs includes acetylcholinesterase inhibitors like donepezil [17], anesthetics such as bupivacaine, certain antiarrhythmics like amiodarone, ivabradine, propafenone, and sotalol, as well as antidepressants like citalopram and fluoxetine, antihypertensive medications like diltiazem and verapamil, beta-blockers, and inotropes such as digoxin [16].

Drug interactions are common in patients with cardiovascular conditions and can present significant risks if not properly managed [18,19]. One particularly challenging interaction occurs between digoxin and beta-blockers. In individuals with atrial fibrillation and heart failure, both beta-blockers and digoxin are used to control ventricular rates. However, the combined use of these drugs raises concerns regarding their impact on patient outcomes, as the interaction may lead to increased risk of morbidity and mortality [20]. Therefore, clinicians must carefully consider the potential toxicity of these drugs in patients who are bradycardic or hypotensive [21,22].

The aim of the present case report was to highlight the critical role of recognizing and managing drug–drug interactions between digoxin and beta-blockers, active pharmacological substances extensively utilized in the treatment of cardiovascular conditions such as atrial fibrillation, heart failure, and hypertension. By presenting a case of a paradoxical finding of CHB with atrial flutter precipitated by the combination of drugs utilized, the report emphasizes the need for heightened vigilance in elderly patients with polypharmacy, where such interactions can be life-threatening. The contribution to scientific literature lies in illustrating the complexities of drug combinations in an elderly patient with polypharmacy, emphasizing the importance of optimal management of drug–drug interactions, as seen in the resolution following the withdrawal of antiarrhythmic medication without the need for permanent pacemaker implantation.

## 2. Detailed Case Description

We report the case of an 82-year-old female diagnosed with a CHB. The patient presented to the emergency department of Clinical County Emergency Hospital Bihor following an episode of syncope preceded by moderate vomiting on the day before her presentation. Her past medical history included atrial fibrillation, hypertension, and heart failure. Her chronic home medication regimen was composed of the following: digoxin 0.25 mg given daily 5 days out of 7, metoprolol 50 mg twice a day, lisinopril 10 mg once a day, furosemide 40 mg once a day, and spironolactone 50 mg once a day. The patient had a total body weight of 77 kg, corresponding to a body mass index of 26, which classifies the patient as overweight.

**Figure 1:**
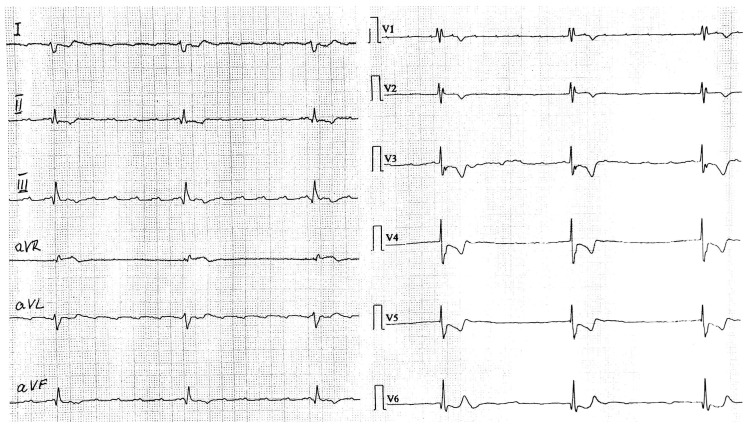
ECG at admission reveals atrial flutter with complete atrioventricular block.

On clinical examination in the emergency department, she was disoriented, with pale, dehydrated skin. The cardiac sounds were bradycardic, and a holosystolic murmur grade 4/6 was audible over the mitral valve area. The ECG at admission (Figure 1) revealed atrial flutter and infra-hisian complete AV block (the escape rhythm with right bundle branch block morphology) with a ventricular rate of 35 beats/min, which was completely different in comparison to a previous ECG of the patient that showed atrial fibrillation with complete left bundle branch block.

Laboratory findings at admission showed mild hyperkalemia (serum potassium = 4.90 mmol/L), slightly elevated gamma-glutamyl transferase (GGT = 68 UI/L), a moderate increase in blood urea (40 mg/dL), and an elevated white blood cell count (WBC = 16.4 × 10^3^/mm^3^) that decreased to the normal range within a few days and was interpreted as being due to dehydration. The neurological examination ruled out any acute pathology. Echocardiography showed a normal left ventricular ejection fraction (50%), a mildly dilated left atrium, and severe mitral regurgitation.

**Figure 2:**
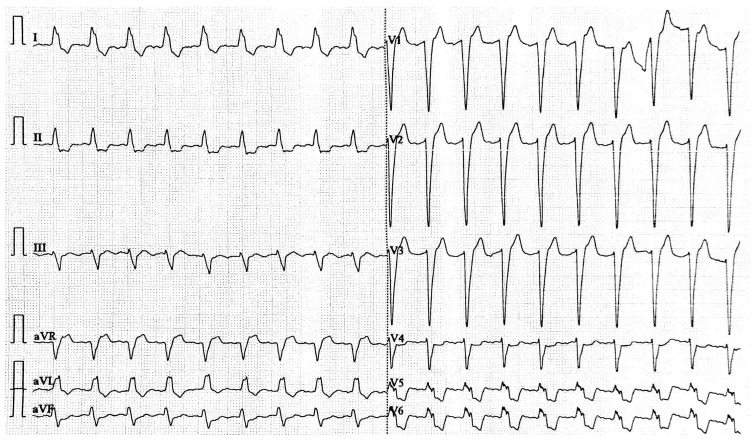
The ECG recording shows atrial fibrillation with a high ventricular rate.

Considering that the combination of digoxin and the beta-blocker was responsible for the new onset of atrial flutter with AV block, the antiarrhythmic therapy was withheld. The presence of mild hyperkalemia could have been a contributing factor. Other potential causes of AV block were excluded. As the patient experienced short, repetitive episodes of non-sustained ventricular tachycardia, a temporary transvenous pacemaker was placed. By the third day after admission, the AV conduction was re-established, the patient’s heart rate increased to 120–150 beats/min, and the ECG showed atrial fibrillation with a high ventricular rate and left bundle branch block (Figure 2).

**Figure 3:**
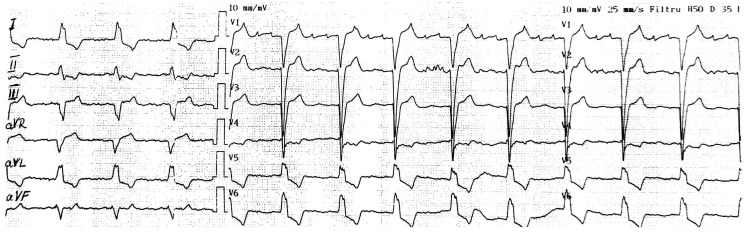
The ECG recording shows atrial flutter with 3:1 AV conduction block.

**Figure 4:**
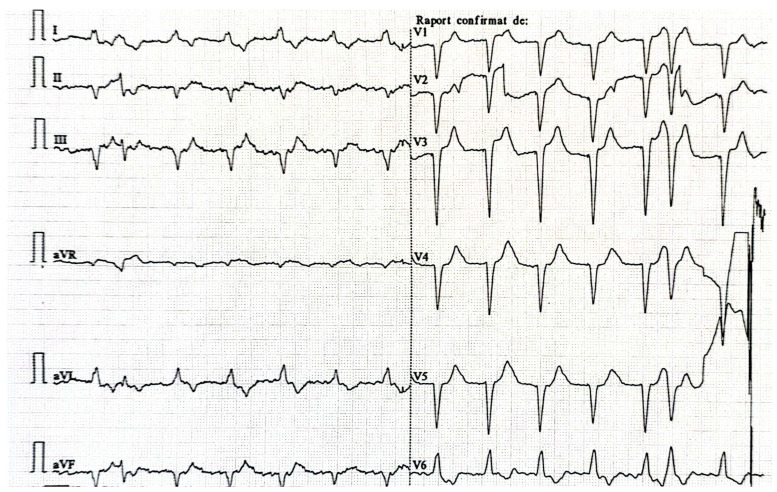
ECG at discharge reveals atrial fibrillation with a moderate heart rate.

A selective β1-blocker (i.e., bisoprolol) was initiated with a progressive increase in dosage until the heart rate decreased. Firstly, the ECG showed atrial flutter with 3:1 conduction (Figure 3), but after one day, the ECG revealed atrial fibrillation with a heart rate of 80/min (Figure 4).

Medical treatment during hospitalization, in addition to antiarrhythmic therapy, included low-molecular-weight heparin, loop diuretics, and normal saline solutions. After the patient’s potassium serum level decreased to a normal range, an angiotensin-converting enzyme inhibitor was added to the medication (i.e., lisinopril). The renal function was closely monitored, and as it showed no pathological changes, it was considered that the association of spironolactone to her treatment would be safe. The outcome was positive, with hemodynamic and electrical stability obtained within a few days, and the patient was discharged with the following medication regimen: apixaban 5 mg 1-0-1, bisoprolol 5 mg 1-0-0, furosemide/spironolactone 20/50 mg 1-0-0, and lisinopril 10 mg 1-0-0.

A 2-month follow-up showed no other AV conduction disturbances, and the AV rate was maintained in normal range.

Possible causes of CHB are intrinsic atrioventricular node disease, myocardial infarction, myocarditis, electrolyte imbalances, hypoxia and certain drugs such as beta-blockers, digitalis, calcium channel blockers, and amiodarone.

In the above reported data, the particularities were the association of CHB to atrial flutter and the rapid resolution of the CHB after discontinuing antiarrhythmic treatment. The combination of digoxin with a beta-blocker and mild hyperkalemia may explain this. The patient tolerated the beta-blocker treatment alone for heart rate control well.

The study was conducted with the approval of The Ethics Committee of the Oradea County Emergency Clinical Hospital, Romania, under reference number 35972/21.11.2024, and in accordance with the ethical principles outlined in the World Medical Association Declaration of Helsinki for medical research involving human participants [23]. Written informed consent was obtained from the patient for the presentation of the present clinical case.

## 3. Discussion

The clinical manifestations and severity of AV blocks depend on the degree of conduction impairment and vary among individuals. First-degree AV block is typically asymptomatic. In contrast, second-degree AV block may range from asymptomatic cases to those involving symptoms such as dizziness, chest pain, palpitations, nausea, or episodes of syncope. Third-degree AV block presents similar symptoms but tends to be more severe due to significant bradycardia [24].

The association of CHB to atrial flutter is a very uncommon condition [25]. The occurrence of CHB in conjunction with atrial flutter is potentially linked to underlying pathological changes in the atrial muscle, including the internodal pathways [26]. When a patient experiences syncope along with paroxysmal supraventricular tachycardia and bundle branch block, it is advisable for clinicians to explore the possibility of an electrophysiological assessment. This evaluation should focus on a comprehensive examination of the conduction system between the atria and the His bundle, incorporating a range of pacing strategies for both atrial and ventricular sites, as well as the use of pharmacological agents for challenge testing [27]. Although at first glance at the ECG the diagnosis would have been of atrial flutter with high-grade conduction block (6:1, highly suggestive of digoxin toxicity), the new QRS pattern of the right bundle branch block (in comparison to the pre-existent left bundle branch block) was indicative of an escape ventricular rhythm. As the condition resolved after antiarrhythmic therapy discontinuation, the cause was considered evident, and there was no need to perform an electrophysiological study.

Initial treatment for symptomatic bradycardia adheres to the advanced cardiac life support guidelines, with atropine as the primary intervention. If atropine fails to resolve symptoms, epinephrine or dopamine is considered. For cases of third-degree AV block unresponsive to medication, transcutaneous pacing is the preferred rapid intervention, ensuring both mechanical and electrical capture. In the absence of success, transvenous pacing becomes necessary. Notably, in instances of drug toxicity, pacing alone is insufficient without addressing the underlying cause [2].

The evaluation of a patient presenting with symptoms of syncope aims to establish a clear diagnosis and assess the severity of the cardiovascular risk. This involves a detailed medical history and physical examination, focusing on key factors: age, as AV blocks are more prevalent in elderly individuals; family history, to identify cases of sudden cardiac death or hereditary cardiac conditions; and personal history of heart diseases and current medications, such as antiarrhythmics, calcium channel blockers, beta-blockers, or digoxin, which may exacerbate bradycardia [2,28].

The cardiovascular comorbidities of our patient necessitated a complex therapeutic regimen involving a positive inotropic agent (e.g., digoxin), a beta-blocker (e.g., metoprolol), an ACE inhibitor (e.g., lisinopril), and two diuretics, a loop diuretic (e.g., furosemide) and a potassium-sparing diuretic (e.g., spironolactone). Among these treatments, digoxin holds a particularly critical role due to its narrow therapeutic index, requiring meticulous monitoring to avoid complications [29].

Although digoxin’s use in managing heart failure and atrial fibrillation has declined, its clinical relevance persists, with over 1.5 million prescriptions issued in 2021 [30]. The drug acts by inhibiting the Na^+^-K^+^-ATPase enzyme on cardiac cell membranes, enhancing myocardial contractility and improving left ventricular ejection fraction in heart failure patients [31,32].

Given the slim margin between therapeutic and toxic doses, the careful safety assessment of digoxin is compulsory. Toxicity symptoms range from confusion and seizures to fatigue and muscle weakness [21], some of which were observed in the present case. Severe toxic effects can result in chronic arrhythmias, such as atrioventricular blocks or ventricular tachycardia [32].

Similarly, beta-blockers, another integral component of the patient’s treatment, carry a risk of inducing bradycardia associated with drug interactions, further emphasizing the need for careful management to prevent adverse cardiac events [33,34].

The clinical findings indicating a CHB prompted an urgent assessment, revealing a drug interaction between metoprolol and digoxin. The scientific literature supports the association of these medications with bradycardia and atrioventricular block [21,35]. These risks are well-documented in case reports focusing individually on digoxin [32,36] and beta-blockers as causative agents of bradycardia [37].

Polypharmacy is more prevalent among the elderly, which increases the risk of interactions among the drugs used [38]. Managing polypharmacy-induced drug interactions is particularly challenging. Online tools for drug interaction checking, such as databases rich in interaction data, are essential for the timely identification of risks. These tools are especially critical in patients on multiple medications, as their complexity increases the probability of adverse interactions [39].

Beta-blockers have also been implicated in interactions leading to CHB when combined with other drugs, such as the antiepileptic lacosamide [40]. Consequently, thorough monitoring of regimens involving beta-blockers or digoxin, especially in patients with chronic conditions, is vital to avoid severe drug–drug interactions.

**Figure 5:**
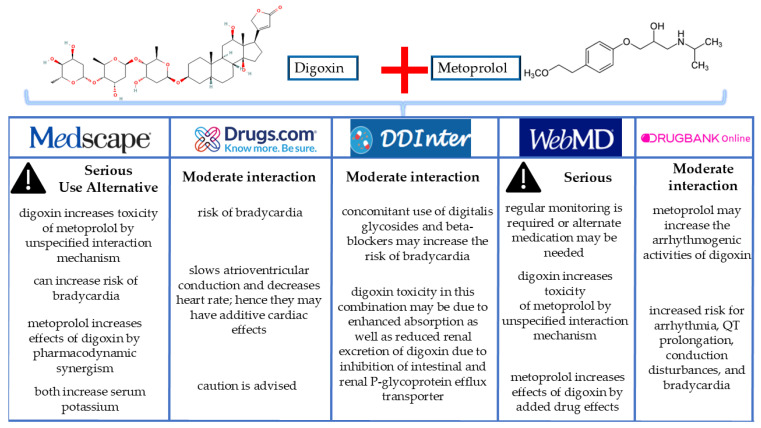
Online interaction checkers showing results for the combination of digoxin with metoprolol.

Verification of the digoxin and metoprolol interaction was performed using online resources like Medscape [41], Drugs.com [42], WebMD [43], DrugBank [44], and DDInter [45]. These platforms categorized the interaction as moderate to serious, with compact and relatively uniform risk and management information. Figure 5 illustrates the results from these tools in evaluating potential drug–drug interactions.

In response to the patient’s clinical presentation, digoxin was discontinued, and metoprolol was substituted with bisoprolol, a selective beta-blocker. Bisoprolol, while effectively reducing heart rate, offers a longer half-life compared to metoprolol, ensuring a more consistent rate control and minimizing the risk of abrupt heart rate fluctuations. This feature enhances its safety profile, particularly in patients prone to significant heart rate variability [46].

**Figure 6:**
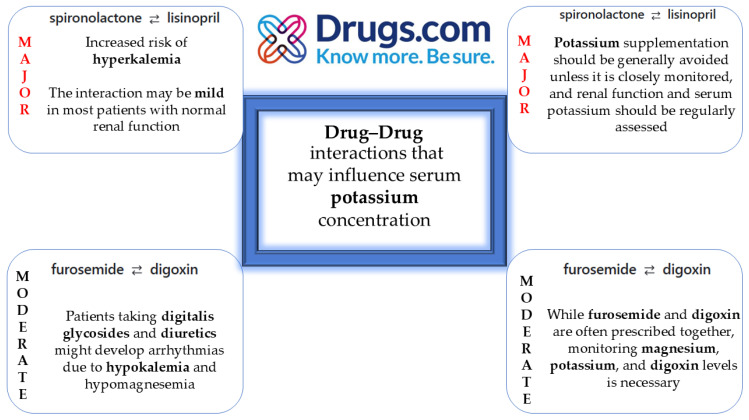
Drug interactions affecting potassium concentrations.

Figure 6 includes the selection of potential drug interactions that may impact serum potassium concentration, with particular implications for cardiac function, whether in the context of hyperkalemia or hypokalemia. For this selection, all medications used by the patient at the time of hospital admission were included, utilizing the drug interaction checker module from drugs.com, which allows for the simultaneous evaluation of all active substances used by the patient. In the context of this case report, the importance of assessing and managing serum potassium levels is emphasized, especially in the context of polypharmacy in patients with cardiovascular diseases.

Within this framework, the impact of serum potassium concentrations on patient management was carefully evaluated in the present case. The values indicated a mild hyperkalemia (4.90 mmol/L), probably due to dehydration and the concomitant use of a potassium-sparing diuretic and an angiotensin-converting enzyme inhibitor. The renal function and serum levels of potassium were carefully monitored, without raising problems in patient management. As the patient had heart failure with preserved ejection fraction, therapy at discharge included spironolactone, a potassium-sparing diuretic, and lisinopril, which together can lead to increases in serum potassium levels [47], but they were added to the treatment regimen only after having evidence of normal renal function and the achievement of a normal potassium serum level. The use of furosemide, a loop diuretic, in the patient’s multiple drug regimen accentuates potassium ion excretion and possibly counterbalances excess potassium ion retention secondary to the association of spironolactone with lisinopril [48].

Continuous monitoring is crucial in clinical contexts involving electrolyte imbalances, as potassium levels directly impact cardiac function. Hyperkalemia, a frequent electrolyte disturbance, poses a risk of severe cardiac conduction abnormalities, including advanced atrioventricular blocks like second- and third-degree blocks [49,50].

**Figure 7:**
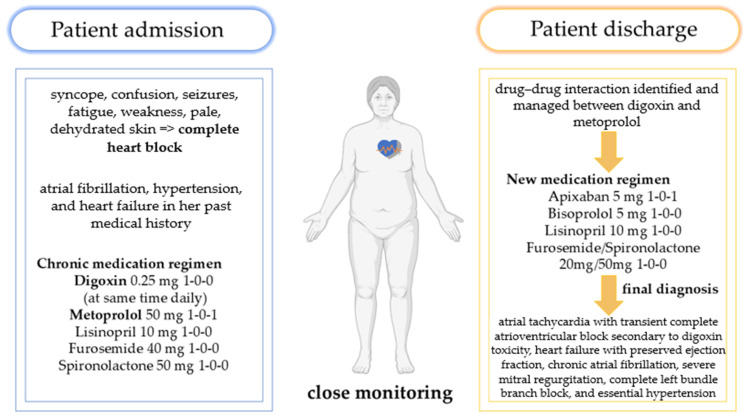
Revised therapeutic regimen based on drug interaction identification and management.

Figure 7 depicts the revised therapeutic plan for this patient, highlighting the adjustments that led to stabilization of the patients’ clinical signs and symptoms.

Research has shown that the narrow therapeutic window of digoxin, coupled with its nonspecific signs and symptoms of toxicity, presents considerable challenges for clinicians in diagnosing toxicity accurately and determining appropriate treatment strategies for their patients [30]. However, management strategies may differ depending on the clinical context. While in most cases, discontinuing digoxin is recommended, particularly when patients exhibit signs of toxicity, there are exceptions [51].

In life-threatening scenarios, such as when patients experience severe arrhythmias or significant conduction disturbances, the use of pacemakers may be indicated to stabilize heart function. The implementation of cardiac pacing demands careful consideration. Although it can be essential and helpful for certain individuals, it should be carried out only in situations where digoxin immune Fab is unavailable and exclusively by qualified clinicians [30].

The results of a similar case report concluded that an increased serum level of digoxin may suggest the need for treatment with digoxin-specific antibody fragments, although the criteria for their use are not uniform. The impact of an implanted pacemaker in patients experiencing digoxin toxicity remains unclear [52]. Additionally, when life-threatening digitalis toxicity is suspected, the administration of digoxin-specific antibodies may be considered as part of the treatment strategy, particularly in cases involving severe or refractory toxicity [51].

The therapeutic management of cardiovascular conditions becomes even more complicated in the context of polypharmacy, where multiple drug interactions can occur. In a case report it has been demonstrated that combining digoxin with certain diuretics, such as furosemide, can lead to significant drug–drug interactions, contributing to digitalis toxicity. In such cases, elevated serum digoxin levels often result from hypokalemia induced by furosemide, which exacerbates the toxic effects of digoxin [53]. Another case report demonstrated that in the context of different types of arrhythmias, discontinuing digoxin is a crucial step, and managing electrolyte imbalances, especially potassium levels, is essential [54].

The conclusion of an additional case report was that, in cases of suspected digoxin toxicity, patients should receive supportive care, which includes cessation of the medication and the potential administration of digoxin-specific antibody fragments. Furthermore, kidney function evaluation is essential for predicting drug toxicity [55].

Interestingly, despite the potential risks of digoxin toxicity, the addition of beta-blockers to digoxin therapy in patients hospitalized with decompensated heart failure has been shown to offer substantial clinical benefits. While digoxin alone, when administered at discharge, is associated with an increased risk of both 1- and 10-year mortality, the concurrent use of beta-blockers significantly reduces this risk. This combination therapy improves long-term survival outcomes, highlighting the complex, yet potentially beneficial role of digoxin when used alongside other agents in specific clinical settings [56].

Therefore, it is imperative to assess the entire clinical and paraclinical context when managing patients on digoxin, particularly in polypharmacy scenarios. Clinicians must be aware of the potential for paradoxical clinical states, such as complete heart block and atrial flutter, which may arise due to drug–drug interactions, as observed in the present case. Timely and evidence-based management, including the discontinuation of digoxin, pacemaker implantation in severe cases, and the use of digoxin-specific antibodies in cases of severe toxicity, remains critical in ensuring optimal patient outcomes.

## 4. Conclusions

The present case report underscores the importance of identifying and managing drug–drug interactions, particularly between digoxin and beta-blockers, in cardiovascular treatment. This case is further complicated by the patient’s rare and paradoxical clinical state, exhibiting both CHB and atrial flutter simultaneously.

The medications used, frequently prescribed for conditions such as atrial fibrillation, heart failure, and hypertension, can pose significant risks when used together. As demonstrated in this patient, their combination led to CHB, a potentially life-threatening complication. The case emphasizes the reversibility of AV block upon discontinuation of digoxin, highlighting the critical need for tailored therapeutic adjustments and vigilant monitoring to prevent adverse outcomes in clinical practice.

Online drug–drug interaction checker tools may be highly important for identifying potential interactions, especially in patients with polypharmacy or complex conditions. Their utility is critical in ensuring safe prescribing practices. Consistent and thorough monitoring remains indispensable for patients on medications with a narrow therapeutic index, like digoxin.

## Data Availability

The raw data supporting the conclusions of this article will be made available by the authors on request.

## Abbreviations

The following abbreviations are used in this manuscript:

AV	Atrioventricular
CHB	Complete heart block
GGT	Gamma-glutamyl transferase
WBC	White blood cell
ECG	Electrocardiogram

## References

[1] Vogler J., Breithardt G., Eckardt L. (2012). Bradyarrhythmias and Conduction Blocks. Rev. Esp. Cardiol. (Engl. Ed.).

[2] Tagarakis G., Gheni A., Hashim H.T. (2023). Complete Heart Block (CHB). Clinical and Surgical Aspects of Congenital Heart Diseases.

[3] Epstein A.E., DiMarco J.P., Ellenbogen K.A., Estes N.A.M., Freedman R.A., Gettes L.S., Gillinov A.M., Gregoratos G., Hammill S.C., Hayes D.L. (2008). ACC/AHA/HRS 2008 Guidelines for Device-Based Therapy of Cardiac Rhythm Abnormalities: A Report of the American College of Cardiology/American Heart Association Task Force on Practice Guidelines (Writing Committee to Revise the ACC/AHA/NASPE 2002 Guideline. J. Am. Coll. Cardiol..

[4] Kashou A.H., Goyal A., Nguyen T., Ahmed I., Chhabra L. Atrioventricular Block. https://www.ncbi.nlm.nih.gov/books/NBK459147/.

[5] Merchant F.M., Hoskins M.H., Musat D.L., Prillinger J.B., Roberts G.J., Nabutovsky Y., Mittal S. (2017). Incidence and Time Course for Developing Heart Failure With High-Burden Right Ventricular Pacing. Circ. Cardiovasc. Qual. Outcomes.

[6] Barra S.N.C., Providência R., Paiva L., Nascimento J., Marques A.L. (2012). A Review on Advanced Atrioventricular Block in Young or Middle-Aged Adults. Pacing Clin. Electrophysiol..

[7] Sundhu M., Yildiz M., Syed M., Shah B., Gul S., Afzal O., Castle L. (2017). Clinical Characteristics and Outcomes of Patients with Ischemic and Non-Ischemic Complete Heart Block. Cureus.

[8] Harikrishnan P., Gupta T., Palaniswamy C., Kolte D., Khera S., Mujib M., Aronow W.S., Ahn C., Sule S., Jain D. (2015). Complete Heart Block Complicating ST-Segment Elevation Myocardial Infarction. JACC Clin. Electrophysiol..

[9] Natsheh A., Shimony D., Bogot N., Nesher G., Breuer G.S. (2019). Complete Heart Block in Lupus. Lupus.

[10] Aksu U., Lazoglu Z., Kalkan K., Topcu S., Tanboga I.H. (2018). A Life-Threatening Condition: Hyperkalemia-Induced Complete Heart Block. Am. J. Cardiol..

[11] Krause P.J., Bockenstedt L.K. (2013). Lyme Disease and the Heart. Circulation.

[12] Knabben V., Chhabra L., Slane M. Third-Degree Atrioventricular Block. https://www.ncbi.nlm.nih.gov/books/NBK545199/.

[13] Zhang J., Liu J., Ye M., Zhang M., Yao F., Cheng Y. (2024). Incidence and Risk Factors Associated with Atrioventricular Block in the General Population: The Atherosclerosis Risk in Communities Study and Cardiovascular Health Study. BMC Cardiovasc. Disord..

[14] Abdel-Daim M.M., Abo-EL-Sooud K., Aleya L., Bungau S.G., Najda A., Saluja R. (2018). Alleviation of Drugs and Chemicals Toxicity: Biomedical Value of Antioxidants. Oxid. Med. Cell. Longev..

[15] Radu A.-F., Bungau S.G., Corb Aron R.A., Tarce A.G., Bodog R., Bodog T.M., Radu A. (2024). Deciphering the Intricate Interplay in the Framework of Antibiotic-Drug Interactions: A Narrative Review. Antibiotics.

[16] Tisdale J.E., Chung M.K., Campbell K.B., Hammadah M., Joglar J.A., Leclerc J., Rajagopalan B., American Heart Association Clinical Pharmacology Committee of the Council on Clinical Cardiology and Council on Cardiovascular and Stroke Nursing (2020). Drug-Induced Arrhythmias: A Scientific Statement from the American Heart Association. Circulation.

[17] Rowland J.P., Rigby J., Harper A.C., Rowland R. (2007). Cardiovascular Monitoring with Acetylcholinesterase Inhibitors: A Clinical Protocol. Adv. Psychiatr. Treat..

[18] Mohamed Ariff A.M., Abd Hadi H., Win N.T., Thoulath M.I., Aktifanus A.T.J., Mutaya S.M., Nalliah S., Mohamed Yusof A.K. (2020). AHMAD Prevalence of Drug to Drug Interactions in Critical Cardiac Patients. Eur. Heart J..

[19] Akbar Z., Rehman S., Khan A., Khan A., Atif M., Ahmad N. (2021). Potential Drug–Drug Interactions in Patients with Cardiovascular Diseases: Findings from a Prospective Observational Study. J. Pharm. Policy Pract..

[20] Lam S.H.M., Romiti G.F., Olshansky B., Chao T.-F., Huisman M.V., Lip G.Y.H. (2024). Combination Therapy of Beta-Blockers and Digoxin Is Associated with Increased Risk of Major Adverse Cardiovascular Events and All-Cause Mortality in Patients with Atrial Fibrillation: A Report from the GLORIA-AF Registry. Intern. Emerg. Med..

[21] Palatnick W., Jelic T. (2020). Calcium Channel Blocker and Beta Blocker Overdose, and Digoxin Toxicity Management. Emerg. Med. Pract..

[22] Fauchier L., Grimard C., Pierre B., Nonin E., Gorin L., Rauzy B., Cosnay P., Babuty D., Charbonnier B. (2009). Comparison of Beta Blocker and Digoxin Alone and in Combination for Management of Patients with Atrial Fibrillation and Heart Failure. Am. J. Cardiol..

[23] World Medical Association Declaration of Helsinki (2014). Ethical Principles for Medical Research Involving Human Subjects. J. Am. Coll. Dent..

[24] Kusumoto F.M., Schoenfeld M.H., Barrett C., Edgerton J.R., Ellenbogen K.A., Gold M.R., Goldschlager N.F., Hamilton R.M., Joglar J.A., Kim R.J. (2019). 2018 ACC/AHA/HRS Guideline on the Evaluation and Management of Patients With Bradycardia and Cardiac Conduction Delay: A Report of the American College of Cardiology/American Heart Association Task Force on Clinical Practice Guidelines and the Heart Rhyth. Circulation.

[25] Hussain M., Awuah D., Deliwala S., Alkotob M.L., Seedahmed E., Bachuwa G. (2022). The Management of Acute Onset Complete Heart Block and Atrial Flutter in a Patient with COVID-19. Eur. J. Case Reports Intern. Med..

[26] Yamashita T., Murakawa Y., Ajiki K., Omata M. (1997). Incidence of Induced Atrial Fibrillation/Flutter in Complete Atrioventricular Block. A Concept of “atrial-Malfunctioning” Atrio-Hisian Block. Circulation.

[27] Georger F., De Roy L., Sorea C., Albenque J.-P., Boveda S., Belhassen B. (2015). Unusual Mechanism of Complete Atrioventricular Block Following Atrial Flutter Ablation. HeartRhythm Case Rep..

[28] Jayaprasad N., Johnson F., Venugopal K. (2006). Congenital Complete Heart Block and Maternal Connective Tissue Disease. Int. J. Cardiol..

[29] Gona S.R., Rosenberg J., Fyffe-Freil R.C., Kozakiewicz J.M., Money M.E. (2023). Review: Failure of Current Digoxin Monitoring for Toxicity: New Monitoring Recommendations to Maintain Therapeutic Levels for Efficacy. Front. Cardiovasc. Med..

[30] Hack J.B., Wingate S., Zolty R., Rich M.W., Hauptman P.J. (2025). Expert Consensus on the Diagnosis and Management of Digoxin Toxicity. Am. J. Med..

[31] Li H., Zhang C.-H., Liu M.-W. (2024). A Case of Digoxin Intoxication Caused by Short-Term Massive Overdose: Case Report. Medicine (Baltimore).

[32] Ravikumar R.H., Pegu B., Bansal H., Soni K.D. (2024). Falling into Complexity: A Case of Digitalis-Induced Fall, Trauma, Symptomatic Bradycardia, and Syncope. J. Fam. Med. Prim. Care.

[33] Griffiths C., Ioannou A., Dickinson B., Metaxa S., Amin F.R., Mandal A.K.J., Missouris C.G. (2022). Drug-Related Bradycardia Precipitating Hospital Admission in Older Adults: An Ongoing Problem. Eur. J. Hosp. Pharm. Sci. Pract..

[34] Lee J.H., Ryu H.M., Bae M.H., Kwon Y.S., Lee J.H., Park Y., Heo J.-H., Lee Y.S., Yang D.H., Park H.S. (2009). Prognosis and Natural History of Drug-Related Bradycardia. Korean Circ. J..

[35] Fauchier L., Laborie G., Clementy N., Babuty D. (2016). Beta-Blockers or Digoxin for Atrial Fibrillation and Heart Failure?. Card. Fail. Rev..

[36] Nguyen D.N.C., Tran V.N., Yaqub S., Basbayraktar B., Farid A., Sleem M. (2024). Digoxin Toxicity in a Patient with Sick Sinus Syndrome: A Case Complicated by Acute Kidney Injury and Concurrent Amiodarone Use. Chest.

[37] Motoishi H., Uesawa Y., Ishii-Nozawa R. (2024). Evaluation of β-Blocker-Induced Bradyarrhythmia Using an Analysis of the Japanese Adverse Drug Event Report Database. Biol. Pharm. Bull..

[38] Gheorghe G., Toth P.P., Bungau S., Behl T., Ilie M., Stoian A.P., Bratu O.G., Bacalbasa N., Rus M., Diaconu C.C. (2020). Cardiovascular Risk and Statin Therapy Considerations in Women. Diagnostics.

[39] Xiong G., Yang Z., Yi J., Wang N., Wang L., Zhu H., Wu C., Lu A., Chen X., Liu S. (2022). DDInter: An Online Drug-Drug Interaction Database towards Improving Clinical Decision-Making and Patient Safety. Nucleic Acids Res..

[40] Lachuer C., Corny J., Bézie Y., Ferchichi S., Durand-Gasselin B. (2018). Complete Atrioventricular Block in an Elderly Patient Treated with Low-Dose Lacosamide. Cardiovasc. Toxicol..

[41] Medscape Interactions Checker Drug Interactions Between Digoxin and Metoprolol. https://reference.medscape.com/drug-interactionchecker.

[42] Drugs.Com Interactions Checker Drug Interactions between Digoxin and Metoprolol. https://www.drugs.com/interactions-check.php?drug_list=883-0,1615-0.

[43] WebMD Interactions Checker Drug Interactions between Digoxin and Metoprolol. https://www.webmd.com/interaction-checker/default.htm.

[44] DrugBank Interactions Checker Drug Interactions between Digoxin and Metoprolol. https://go.drugbank.com/drug-interaction-checker#results.

[45] DDInter Interactions Checker Drug Interactions between Digoxin and Metoprolol. https://ddinter.scbdd.com/inter-checker/.

[46] Dasbiswas A., Reddy P.K.M., Gajapati V., Rawat C.R., Dharmadhikari A. (2021). Efficacy & Tolerability of Bisoprolol in Comparison to Metoprolol in Indian Patients with Stage-1 Hypertension: Multicentre, Parallel Group, Open Labelled, Randomised Noninferiority Clinical Study. Eur. Heart J..

[47] Abbas S., Ihle P., Harder S., Schubert I. (2015). Risk of Hyperkalemia and Combined Use of Spironolactone and Long-Term ACE Inhibitor/Angiotensin Receptor Blocker Therapy in Heart Failure Using Real-Life Data: A Population- and Insurance-Based Cohort. Pharmacoepidemiol. Drug Saf..

[48] Lin Z., Wong L.Y.F., Cheung B.M.Y. (2022). Diuretic-Induced Hypokalaemia: An Updated Review. Postgrad. Med. J..

[49] Baratloo A., Haroutunian P., Rouhipour A., Saeed S., Rahmati F. (2014). Hyperkalemia-Induced Complete Heart Block. J. Emerg. Pract. Trauma.

[50] Barold S.S., Herweg B. (2014). The Effect of Hyperkalaemia on Cardiac Rhythm Devices. EP Eur..

[51] Andrews P., Anseeuw K., Kotecha D., Lapostolle F., Thanacoody R. (2023). Diagnosis and Practical Management of Digoxin Toxicity: A Narrative Review and Consensus. Eur. J. Emerg. Med..

[52] Bridwell R.E., Baker K.A., Hoyte C.O., Ng P.C. (2019). Digoxin Toxicity in a Patient with Pacemaker: A Case Report. Cureus.

[53] Patel R., Patel P., Patel N., Gangwani J., Patel D. (2022). Case Report on the Interaction between Furosemide and Digoxin That Caused Digoxin Toxicity. J. Drug Deliv. Ther..

[54] Sengul Bag F., Yalcin M., Vatansev H., Comakli H. (2021). Digoxin’s Interactions with Various Drugs and A Case Report. Eur. J. Toxicol..

[55] Digiovanni-Kinsley S., Duke B., Giovane R., Paisley C. (2021). A Case of Digoxin Toxicity Due to Acute Renal Failure. Cureus.

[56] Katz A., Maor E., Leor J., Klempfner R. (2016). Addition of Beta-Blockers to Digoxin Is Associated with Improved 1- and 10-Year Survival of Patients Hospitalized Due to Decompensated Heart Failure. Int. J. Cardiol..

